# Storage/Turnover Rate of Inorganic Carbon and Its Dissolvable Part in the Profile of Saline/Alkaline Soils

**DOI:** 10.1371/journal.pone.0082029

**Published:** 2013-11-29

**Authors:** Yugang Wang, Zhongyuan Wang, Yan Li

**Affiliations:** 1 State Key Laboratory of Desert and Oasis Ecology, Xinjiang Institute of Ecology and Geography, Chinese Academy of Sciences, Urumqi, Xinjiang, China; 2 University of Chinese Academy of Sciences, Beijing, China; DOE Pacific Northwest National Laboratory, United States of America

## Abstract

Soil inorganic carbon is the most common form of carbon in arid and semiarid regions, and has a very long turnover time. However, little is known about dissolved inorganic carbon storage and its turnover time in these soils. With 81 soil samples taken from 6 profiles in the southern Gurbantongute Desert, China, we investigated the soil inorganic carbon (SIC) and the soil dissolved inorganic carbon (SDIC) in whole profiles of saline and alkaline soils by analyzing their contents and ages with radiocarbon dating. The results showed that there is considerable SDIC content in SIC, and the variations of SDIC and SIC contents in the saline soil profile were much larger than that in the alkaline profile. SDIC storage accounted for more than 20% of SIC storage, indicating that more than 1/5 of the inorganic carbon in both saline and alkaline soil is not in non-leachable forms. Deep layer soil contains considerable inorganic carbon, with more than 80% of the soil carbon stored below 1 m, whether for SDIC or SIC. More importantly, SDIC ages were much younger than SIC in both saline soil and alkaline soil. The input rate of SDIC and SIC ranged from 7.58 to 29.54 g C m^-2^ yr^-1^ and 1.34 to 5.33 g C m^-2^ yr^-1^ respectively for saline soil, and from 1.43 to 4.9 g C m^-2^ yr^-1^ and 0.79 to 1.27 g C m^-2^ yr^-1^respectively for alkaline soil. The comparison of SDIC and SIC residence time showed that using soil inorganic carbon to estimate soil carbon turnover would obscure an important fraction that contributes to the modern carbon cycle: namely the shorter residence and higher input rate of SDIC. This is especially true for SDIC in deep layers of the soil profile.

## Introduction

Soil carbon is the largest carbon pool in the terrestrial biosphere. Globally, carbon stock in soil is approximately twice as large as in the atmosphere and approximately three times that in vegetation [1,2]. Changes in soil carbon storage have long been studied due to its potential to accelerate or mitigate CO_2_ increase in the atmosphere [3-6]. Small losses from this large pool could significantly impact future carbon concentrations in the atmosphere, so the response of soil to global change is of critical importance when assessing climate/carbon cycle feedbacks [7,8]. The reliable assessment of soil carbon stock is of key importance for soil conservation and in mitigation strategies for atmospheric carbon [9]. Therefore, knowledge of the spatial distribution of soil carbon with depth is of great importance for carbon stock accounting and as inputs to hydrological and climate modeling [10]. There is a growing interest in the character of carbon turnover in soil. As the largest carbon storage location in the terrestrial biosphere, even a minor carbon storage change in soil could result in a considerable alteration of atmospheric carbon concentration [11-13]. 

Soil properties play a major role in both the biological and hydrogeological carbon cycle [14]. One important aspect is the relative importance of the soil inorganic carbon (SIC) pool and its dynamics in arid and semiarid regions [15,16]. However, the role of the soil inorganic carbon pool in the greenhouse effect is much less understood in the global carbon cycle [17,18]. Soil consists of various chemical substances, and many elements in soil are dissolvable in water. Soil dissolved inorganic carbon (SDIC), a dynamic part of soil inorganic carbon, influences biogeochemical processes in aquatic and terrestrial environments [19-21]. Dissolved inorganic carbon plays a significant role in the interactions of soils and solution such as hydrolysis, hydration, solution, oxidation, carbonation and so on [22], thereby serving as a sensitive indicator of shifts in soil processes [23,24]. Understanding the significance of SDIC in soil processes can help in developing strategies to mitigate atmospheric carbon concentrations [25]. 

Soil inorganic carbon (SIC) is the most common form of carbon in arid and semiarid regions, and has a long turnover time [26]. Little is known, however, about SDIC storage and its turnover time in soils. In arid areas, soils generally have saline or alkaline characteristics [27]. Saline soil refers to a soil that contains sufficient soluble salts to impair its productivity, and have an electrical conductivity (EC) of the saturation soil extract of more than 4 dS/m at 25°C. Similarly, alkaline soils are also defined in terms of impaired productivity caused by exchangeable sodium, which usually have a high pH and low EC [28]. Soils often interact with rainfall, irrigation, etc. The soluble salts that occur in soils consist mostly of various proportions of anions such as sulfate, chloride, and biocarbonate, and the cations such as calcium, sodium and magnesium, etc. Transport directly as SDIC in soil through the hydrologic cycle is an important component of global carbon budgets [23,29], but there is considerable uncertainty about the amount and accumulation rate of SDIC contained in soil. 

To our knowledge, only a few studies have focused on procedures of differentiating SDIC from SIC in soil: reliable estimates for SDIC in soils are fewer [30]. In this work, a new leaching procedure has been developed based on the application of carbonate dissolution in water. In the current study, we analyzed SIC distribution in profiles of saline and alkaline soil. The aims were: (1) to effectively unravel the dissolved and non-dissolved carbonate by carbonate dissolution in water; (2) to quantify the distribution of SDIC and SIC content and storage in saline and alkaline soil profiles at different depths to explore the importance of deep soil carbon storage below 1 m; (3) to determine turnover time and accumulation rates of SDIC and SIC in saline and alkaline soil profiles. 

## Materials and Methods

### Study area

This study was conducted at the lower reach of an inland river basin on an alluvial plain in the southern Gurbantongute Desert region, China. Geologically, the area was an alluvial plain and the parent material is not from bedrock weathering. This study focused on soil carbonate storage and turnover time, and the Chinese government encourages research on the soil carbon cycle. Thus, soil sampling in the natural landscape was permitted and no specific permissions were required in this region. We confirmed that soil sampling did not involve any endangered or protected species.

Sampling locations were chosen based on apparent differences in natural vegetation typical of saline and alkaline soils. A Global Positioning System (GPS) was used to record the elevation, longitude, and latitude of the sites. The saline soil sampling site is located at the Fu-Kang Station of the Desert Ecology, Chinese Academy of Sciences (44°17´N, 87°56´E and 475 m a.s.l.), where saline land is widely distributed [31]. The topography in sampling site is generally flat (slope < 1°), and the groundwater table has extended down to a depth of 6 m in recent years. *Tamarix ramosissima* Ledeb is the dominant species (average canopy cover ~ 17%) in this desert phreatophyte shrub community [32]. The cover of herbaceous species (including *Salsola nitraria* Pall., *Suaeda acuminate* Moq. and *Salicornia europaea* Linn.) varies greatly, from 5% to 30% between years [33]. The alkaline soil sampling site (44°22'N, 87°55'E, 448 m a.s.l) is less than 8 km north of the saline soil site, located at the edge of the Gurbantongute Desert. The plant community is dominated by the desert shrub *Haloxylon persicum* Bge and *Haloxylon ammodendron* Bge, and other common species include *Alhagi sparsifolia* Shap, *Calligonum leucocladum* Bge, And *Ceratocarpus arenarius* L, etc. The local climate is arid continental with hot summers and cold winters. Annual precipitation averages 163 mm and annual pan evaporation is 1780–2460 mm.

### Soil sampling and analysis

Soil samples were obtained from trenches down to 6 and 9 m until the groundwater table was reached. Given the indeterminate nature of many soil profiles, it is difficult to define what the maximum depth for soil is. Rooting depth may be a good estimation for soil depth, but leaching of soil organic matter or micro-biomes can go much deeper than rooting depth [25]. Thus, here we consider the depth of soil is down to groundwater. Therefore, soil profiles were sampled from the topsoil to the groundwater table to study on distribution of SIC and SDIC. Soils were sampled from horizon to the groundwater table from trenches that had been mechanically excavated at both sites, and three soil profiles in each site were in under sub-scrub canopy, edge of scrub canopy, and neighboring open plot. The parent materials in the sampling area were alluvium materials. The sampling depth of saline soil was from 0 to 6 m, and 12 samples were collected discretely at the depth of 0 to 0.2 m, 0.2 to 0.4 m, 0.4 to 0.6 m, 0.6 to 0.8 m, 0.8 to 1.0 m, 1.0 to 1.5 m, 1.5 to 2.0 m, 2.0 to 2.5 m, 2.5 to 3.0 m, 3.0 to 4.0 m, 4.0 to 5.0 m and 5.0 to 6.0 m respectively from each profile. The sampling depth of alkaline soil was from 0 to 9 m, and 15 samples were collected discretely at the depth of 0 to 0.2 m, 0.2 to 0.4 m, 0.4 to 0.6 m, 0.6 to 0.8 m, 0.8 to 1.0 m, 1.0 to 1.5 m, 1.5 to 2.0 m, 2.0 to 2.5 m, 2.5 to 3.0 m, 3.0 to 4.0 m, 4.0 to 5.0 m, 5.0 to 6.0 m, 6.0 to 7.0 m, 7.0 to 8.0 m and 8.0 to 9.0 m respectively from each profile. Thus there were 81 soil samples taken from 6 profiles in the southern Gurbantongute Desert, China.

Soil bulk density was determined using a soil corer (strainless steel cylinder of 100 cm^3^ in volume). In the laboratory, each soil sample was air-dried and sieved to 2 mm for chemical analysis. To quantify the age of the soil inorganic carbon and soil dissolved inorganic carbon in the soil profile, conventional ^14^C dating of the SIC and SDIC were performed at the ^14^C Laboratory at Lanzhou University, China (lab code Lug; half-life: 5568 ± 40 yr) [34]. 

We developed an equation to quantify the soil dissolved inorganic carbon (SDIC) based on carbonate dissolution in water and mass-balance. For an individual soil sampling, the equation (1) was used to calculated SDIC (g kg^-1^) 

$$SDIC=P_{}-P_{a}$$ (1)

Where *P* is soil inorganic carbon content (g C kg^-1^), *P*_*a*_ is soil non-dissolved inorganic carbon content when EC _soil-water solution_ = EC _water_. The SIC (*P* value) of the whole soil was determined by the Sherrod et al’s method (2002) [35]. After then, determining *P*_*a*_ is the key to calculate SDIC value. 

To measure *P*_*a*_, according to the methodology and procedures of soil salinity analysis [28], soil and water was mixed at a 1:5 soil-water ratio in a mechanical shaker for 15 minute, and then the soil-water solution was separated and extracted by centrifugation, with the temperature controlled at 25°C. Centrifugal time was 15 minutes to fully separate the soil and solution. Liquid was filtered out after the soil solution was centrifuged, and then the same amount of water was added as in the first step. Leaching process was repeated at least 9 times in five and a half of hours until EC _soil-water solution_ = EC _water_, e.g., when there is no detectable difference between these two values. At this time, we considered that all the dissolved inorganic carbon has been separated from the initial soil. The SIC measured on the fully leached soil by the Sherrod et al’s method (2002) [35] was the P_*a*_ in Eqa.1. With Eqa.1, SDIC was calculated and is defined as leachable inorganic carbon. 

Soil inorganic carbon content and no-dissolved inorganic carbon content were determined by a modified pressure transducer method described by Sherrod et al (2002) [35]. For an individual profile with *k* layers, the equation of Batjes (1996) [36] was used to calculate the amount of inorganic carbon in the whole soil profile:

$$SIC_{d}=\sum_{i=1}^{k}SIC_{i}=\sum_{i=1}^{k}\rho_{i}\times P_{i}\times D_{i}\times (1-S_{i})$$ (2)

$$SIC_{i}=\rho_{i}\times P_{i}\times D_{i}\times (1-S_{i})$$ (3)

Where *k* is the number of horizons, *SIC*_*i*_ is soil inorganic carbon storage (Mg m^-2^), *ρ*_*i*_ is the bulk density (Mg m^-3^), *P*_i_ is soil inorganic carbon content (g C g^-1^) in layer *i*, *D*_*i*_ is the thickness of this layer (m), and *S*_*i*_ is the volume fraction of fragments > 2 mm.

Calculation of the SDIC in the soil profile is based on the functional, mass-balance relationship among bulk density, soil chemical composition, and volume change in relation to the soil parent material. The SDIC was calculated by mass loss of ions as follows [37]:

$$SDIC_{d}=\sum_{i=1}^{k}SDIC_{i}=\sum_{i=1}^{k}\rho_{i}\times D_{i}\times (1-S_{i})\times (P_{i}-P_{ia})$$ (4)

$$SDIC_{i}=\rho_{i}\times D_{i}\times (1-S_{i})\times (P_{i}-P_{ia})$$ (5)

Where *k* is the number of horizons, *SDIC*_*i*_ is the soil dissolved inorganic carbon storage (Mg m^-2^), *ρ*_*i*_ is the bulk density (Mg m^-3^), P_i_ is the soil inorganic carbon content (g C g^-1^) in layer *i*, *D*_*i*_ is the thickness of this layer (m), *S*_*i*_ is the volume fraction of fragments > 2 mm, and *P*_ia_ is the soil non-inorganic carbon content in layer *i*.

### Statistical analysis

Descriptive statistical method was used to process the analytical data in terms of its distribution among the studied parameters. The commercial statistics software package SPSS version 17a for Windows was applied for statistical analyses in the current study. Basic statistical parameters such as mean and standard error were calculated. All data were given as online supporting materials (Table S1 in File S1).

## Results and Discussion

### pH and EC change in soil solution

**Figure 1:**
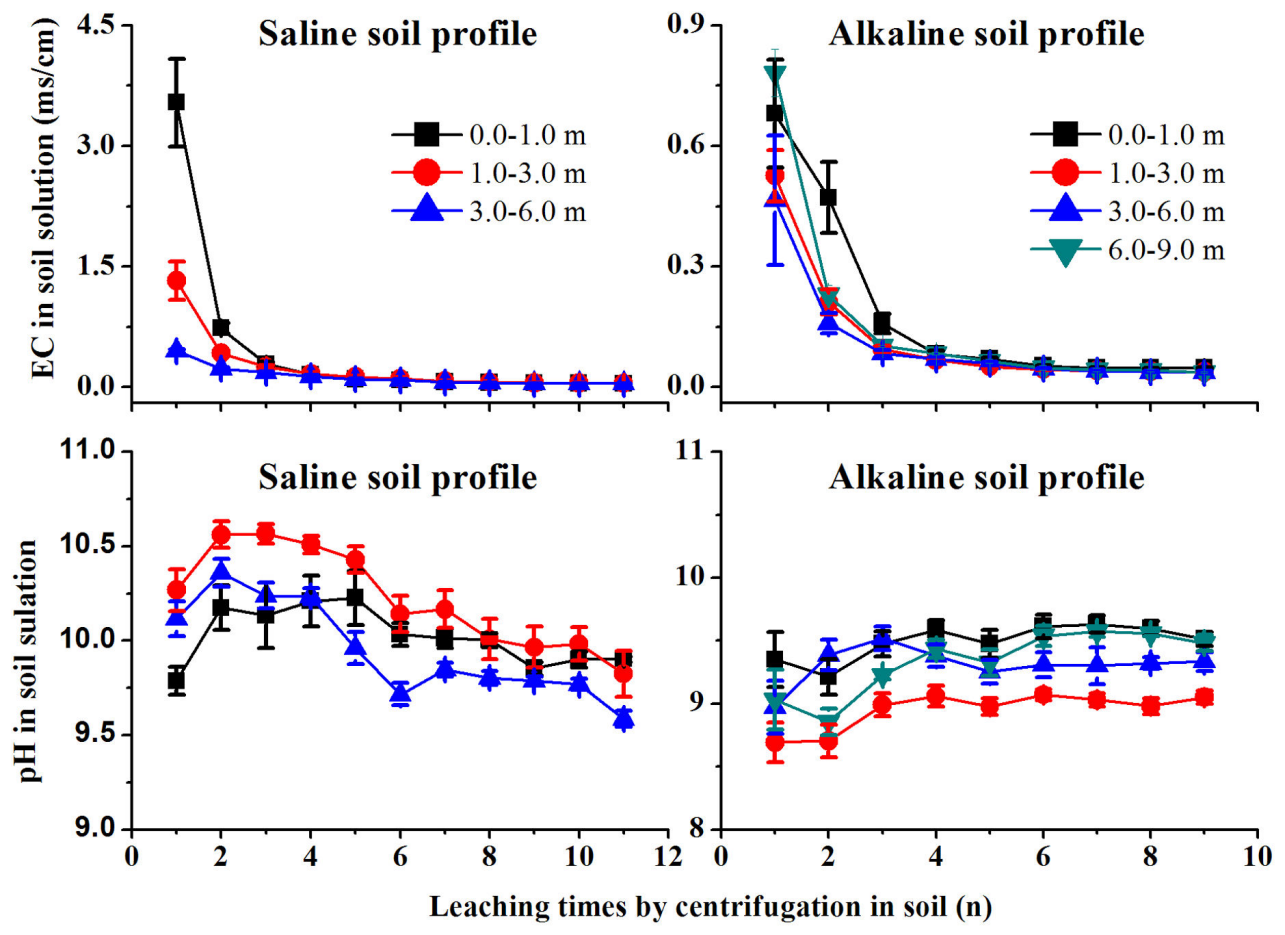
The changes of mean pH and EC values and standard error in the soil-water extracts by centrifugation.

In this study, a new leaching procedure for saline and alkaline soil properties has been developed for carbonate dissolution in water. The proposed method to quantify SDIC is based on the diffusion of carbonate in water, which causes efficient conversion of inorganic carbonates to dissolved ions. This method was applied to separate dissolved and non-dissolved inorganic carbon by mass loss of dissolved carbonate ions in saline and alkaline soils. EC values in the soil-water extract solution decreased with increased leaching times in both saline and alkaline soils (Figure 1), indicating that the dissolved carbonate in soils was leached and secondary carbon as SDIC content occurs in a variety of forms in soil [38]. Because the conversion efficiency of SDIC is dependent upon EC in the soil-water extract solution, soil may be affirmed as having no dissolved carbonate when EC _soil-water solution_ approached EC_water_. Therefore, the EC value revealed different declines in different soil layers whether they were saline or alkaline (Figure 1). With increasing moisture content, pH value may increase or decrease in soil [39]. In the saline soil profile, for the soil-water extracted by centrifugation, with an increase in leaching time, pH values increased at the beginning and then decreased. However, in alkaline soil, pH values decreased first and then increased with leaching except for at depths of 3.0-6.0 m. pH values at depths of 3.0-6.0 m in alkaline soil showed a trend similar to the saline soil (Figure 1).

### SIC and SDIC contents in saline or alkaline soil profiles

**Figure 2:**
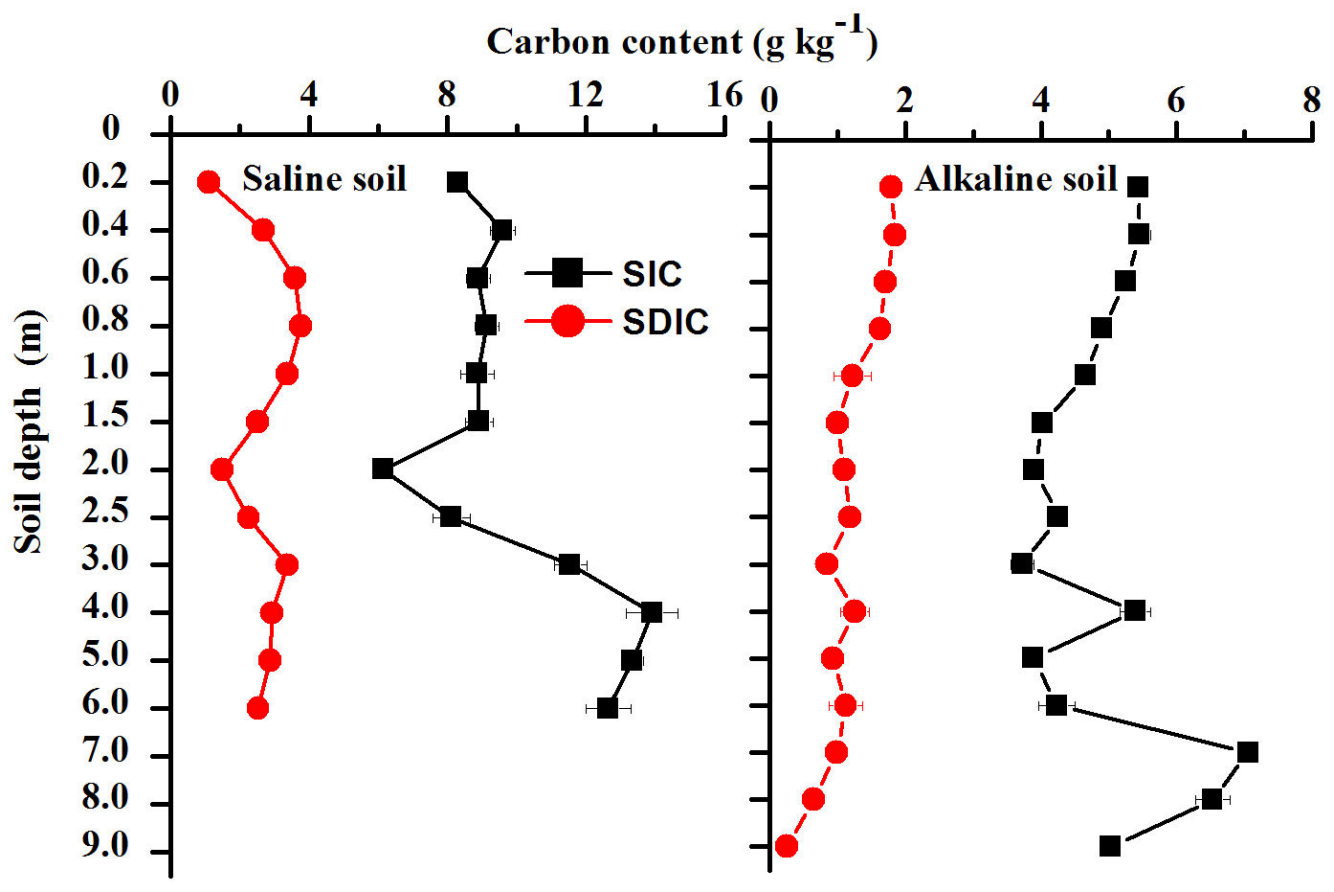
Mean SIC and SDIC content and standard error in the saline and alkaline soil profiles.

SIC and SDIC contents in the saline and alkaline soil profiles are shown in Figure 2. The SIC and SDIC content appear remarkably different between the saline and alkaline soils, and the SIC content was higher than the SDIC content in the same soil layer. Vertical distribution of SIC and SDIC in the soil profiles followed a similar trend. In the saline soil profile, the range in the SIC content of the layers was 6.1-13.9 g/kg, but the SDIC content was 1.5-3.6 g/kg at 6 m depth. Similarly, the SIC was 3.7-6.5 g/kg in the alkaline soil; the SDIC was 0.2-1.9 g/kg. Obviously, the variation of SDIC and SIC contents in the saline soil profile is significantly larger than in the alkaline profile. In addition, average values of the SIC in the saline soil profile at the same depth were higher than in the alkaline soil. The complicated profile distribution for SDIC and SIC may be attributed to soil properties, vegetation type, precipitation, shallow groundwater and so on [40-43]. Vegetation plays an important role in spatial heterogeneity of soil characteristics. Spatial variability of soil characteristics was mainly determined by scrubs [44]. Thus, soils were sampled under sub-scrub canopy, at the edge of scrub canopy, and at the neighboring open plot.

There is evidence that carbon changes over time at both shallow and deep soil horizons can be relatively rapid when management practices are altered, and thus deep soil sampling may be essential in understanding the full impacts of management changes [10]. In studies that seek to determine changes in SIC and SDIC over time, it cannot be assumed that the deep C content does not change, as the production of soluble C compounds in the C pools above might leach into the deeper soil horizons [45,46]. We are aware that solubility of SDIC was relative. The Ca and Mg carbonates are hardly dissolvable in short term, but can be dissolved and leached in long run. Thus the value of SDIC determined in the current study, might have underestimated the SDIC leaching and moving in soil. Lack of data on soil carbon distribution in profiles in different landscapes is one of the major gaps in soil science knowledge [47]. In the natural landscape, there is a wide distribution of saline and alkaline soils in arid regions: our results partially filled this knowledge gap and should help improve global predictions of soil carbon storage.

### SIC and SDIC storage in saline and alkaline soil profiles

**Figure 3:**
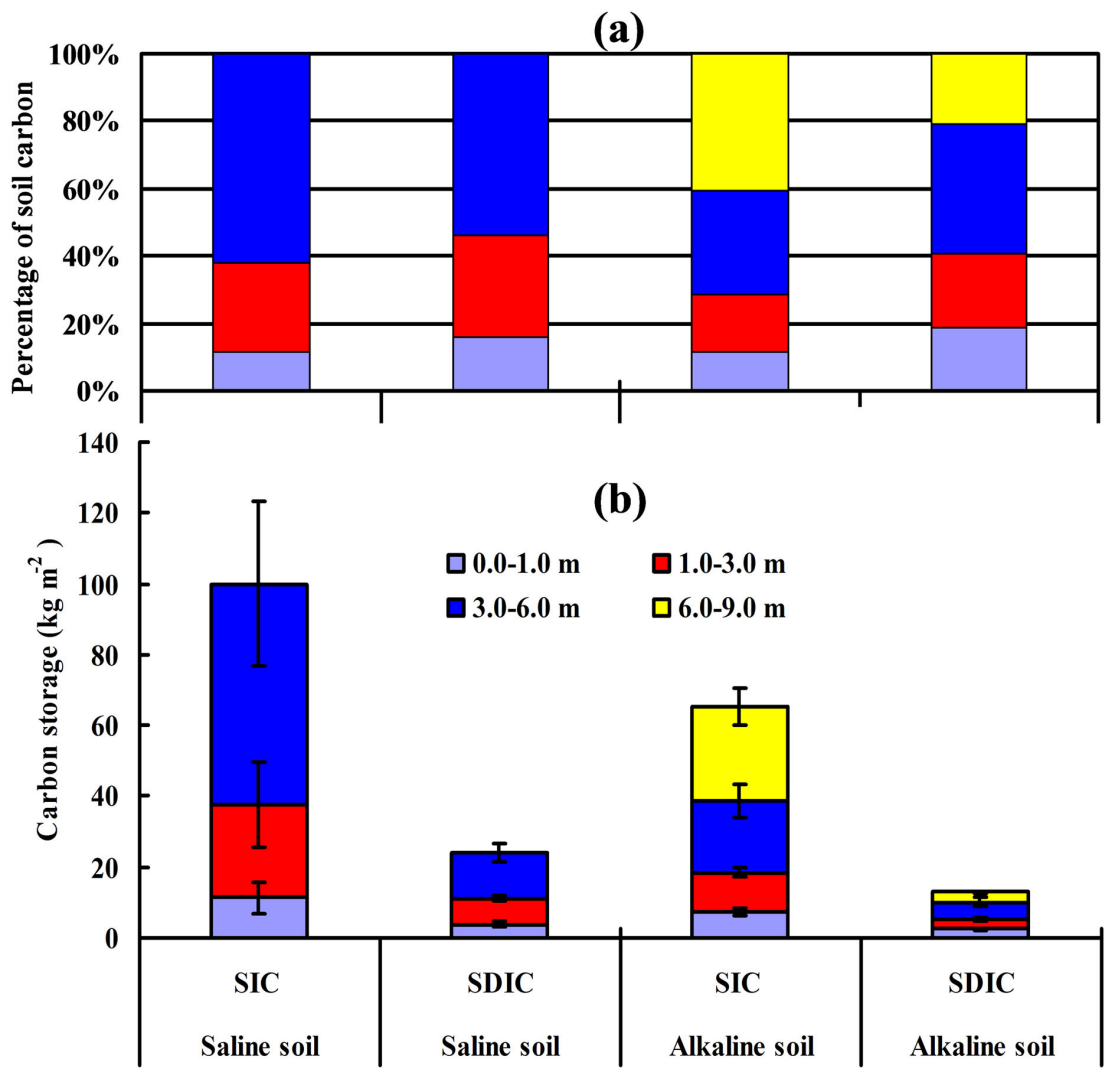
Soil carbon storage at different depths for saline and alkaline soils. Shown in (a) percentage of soil carbon, and (b) mean soil carbon density and standard error.

Figure 3 shows SIC and SDIC storage in layers at depths of 0-1 m, 1-3 m, 3-6 m, and 6-9 m for both saline and alkaline soils. Figure 3(a) gives the percentage of carbon storage at different depths. There was significant SIC and SDIC storage below 1 m depth: SIC and SDIC storage below 1 m depth accounted for more than 80% of that for the soil profile, and more than 50% carbon storage was below 3 m depth. Figure 3(b) shows the distribution of carbon storage at different depths in the soil profile. SIC and SDIC storage were remarkably different between the saline and alkaline soil profiles. The SIC storage from 0-6 m in saline soil (99.97 kg m^-2^) was as high as 2.58 times of that in alkaline soil (38.68 kg m^-2^), and the SDIC storage from 0-6 m in saline soil (24.2 kg m^-2^) was as high as 2.38 times of that in alkaline soil (10.17 kg m^-2^). For the same soil type, SDIC storage was more than 20% of SIC storage, indicating that approximately 1/5 of the inorganic carbon in saline and alkaline soils can be dissolved by water. Clearly, most of this dissolved inorganic carbon can be in a leachable form.

This study gives a first insight into the effect of SIC and SDIC concentration in a whole soil profile. The SIC and SDIC storage indicated that there is significant soil carbon storage below 1.0 m, whether it is mainly SIC in saline soil or SDIC in alkaline soil (Figure 3). There is growing interest in studies on soil carbon storage in deeper profiles, especially the below 1.0 m [10,48,49]. Li et al (2007) [50] estimated that SIC storage below 1 m to full soil profile was approximately 57 % in solonchaks soil and 61% in grey desert soil, and Wang et al (2010) [42] suggested approximately 50% in desert soil. These values were smaller than the values we measured (Figure 3(a)). The discrepancy between these data may be attributed to different soil sampling depth. Most soils in northwest China are well developed, especially in the continental plain of Xinjiang: soil profiles extend far beyond 3 m, and most soil profiles are developed until the groundwater table. The contribution of soil inorganic carbon to total terrestrial carbon storage in China has not been well documented [50]. In addition, data in this study showed that the SIC contains a portion of the SDIC, which suggests that SDIC storage could be more important than SIC storage at the continental scale, especially now as increasing evidence shows that SIC might be dynamic [51,52]. Thus, dissolution of carbonates and leaching in the soil profile may lead to C sequestration by moving carbonates into the groundwater [53]. Obviously, transfer of dissolved inorganic carbon in soil through the hydrologic cycle is an important component of global carbon budgets.

### Dynamics of SIC and SDIC in saline and alkaline soils

**Figure 4:**
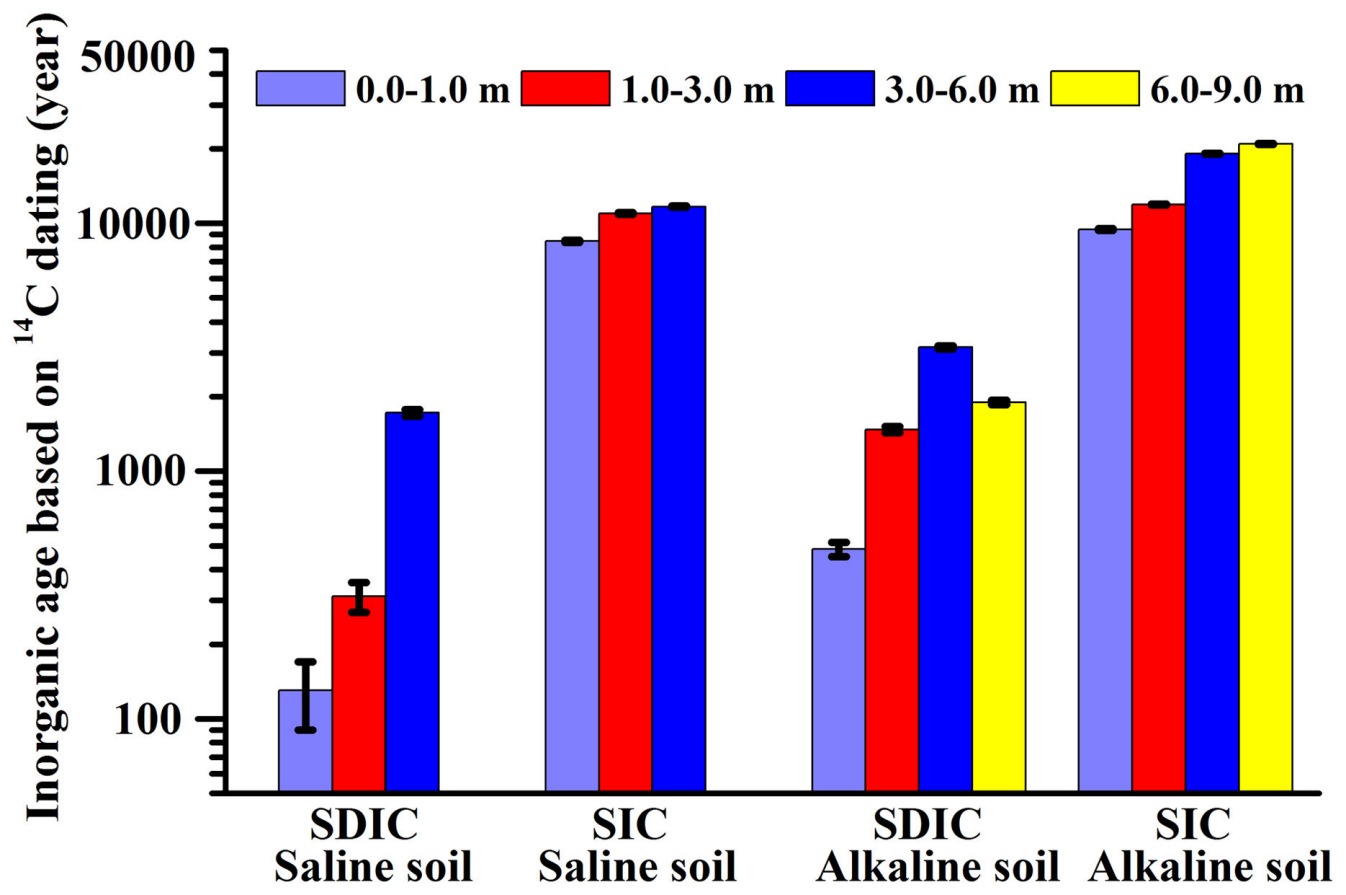
Mean residence times of SIC and SDIC and standard error at different soil layers in the saline and alkaline soil profiles.

**Figure 5:**
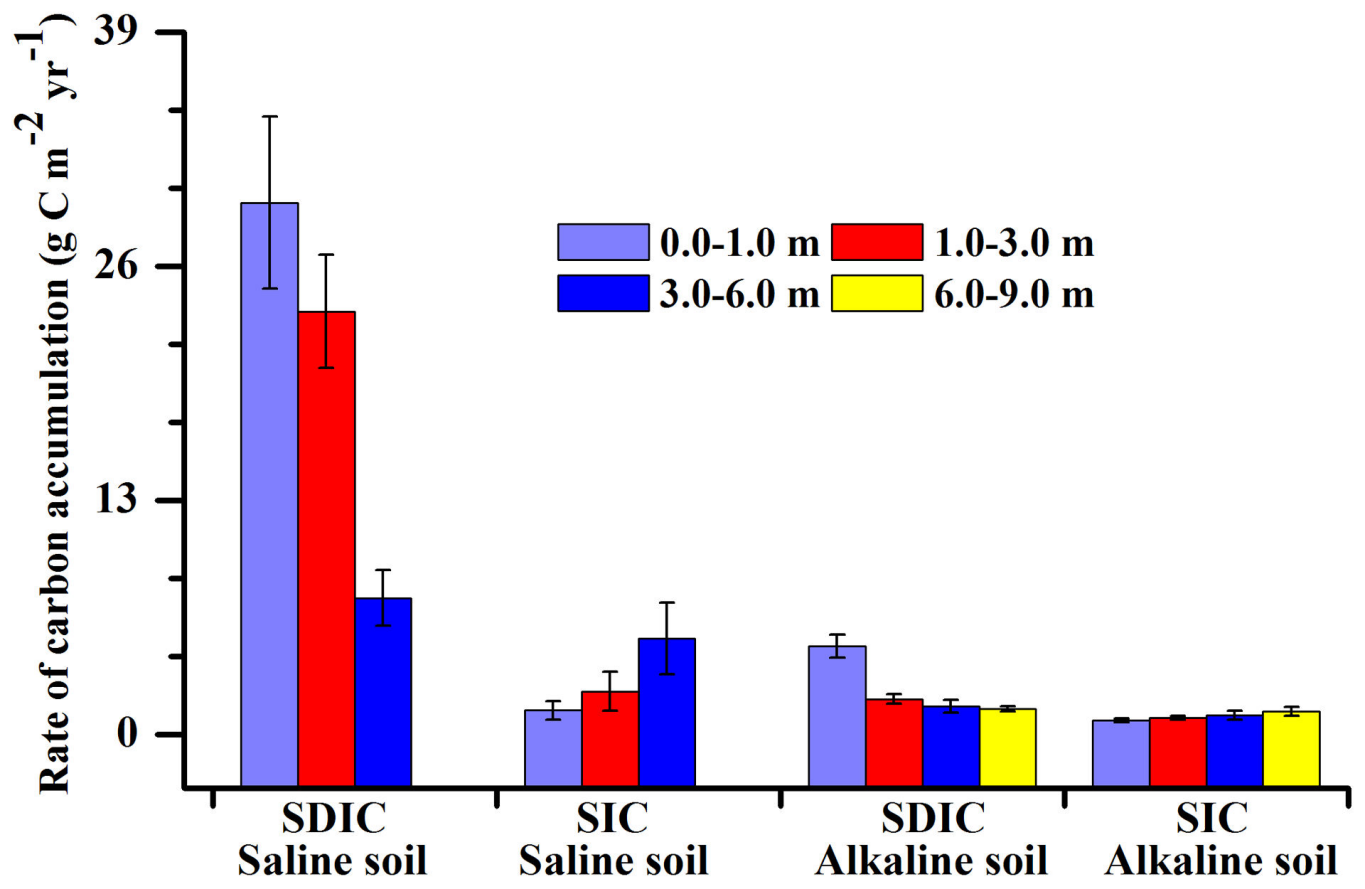
Mean rate of SIC and SDIC accumulation and standard error at different soil layers in the saline and alkaline soil profiles.

Figure 4 shows the mean residence time of carbon in the saline and alkaline soil profiles. The mean residence time of SDIC was significantly shorter than SIC. SDIC ages were younger than 4000 years, but most SIC ages were approximately 10,000 years. For the saline soil profiles, mean residence time of SDIC increased with soil depth from 130 years at 0-1 m depth to 1719 years at 3-6 m, and SIC varied from 8477 years at 0-1 m depth to 11,688 years at 3-6 m depth (Figure 4). The carbon accumulation rate for SDIC is higher than SIC in the same soil layer. The long-term carbon accumulation rate for SDIC ranges from 29.54 at 0-1 m depth to 7.58 g C m^-2^ yr^-1^ at 3-6 m depth, with a decreasing trend with depth. For SIC, the rate was 1.34 at 0-1 m depth to 5.33 g C m^-2^ yr^-1^ at 3-6 m depth, with an increasing trend with depth (Figure 5). For the alkaline soil profile, residence times of SDIC ranged from 480 at 0-1 m depth to 3200 years at 6-9 m depth, and the input rate declined from 4.9 g C m^-2^ yr^-1^ at 0-1 m depth to 1.43 g C m^-2^ yr^-1^ at 6-9 m depth (Figure 4), which may be attributed to an accumulation of secondary carbonates through dissolution of CO_2_ in the surface layer followed by translocation and re-precipitation with Ca^+2^ and Mg^+2^ in the subsoil [54]. SIC ages are between 9000 years and 21,000 years, and the input rate was from 0.79 to 1.27 g C m^-2^ yr^-1^ in soil layer from depths of 0-1 m to 6-9 m (Figure 4). 

The difference rate in carbon accumulation between SDIC and SIC should have been resulted from the difference of their origins. SDIC is mostly dissolvable from soil air [55], while SIC is mostly from atmospheric deposition of calcium [26]. In a similar area, Schlesinger (1985) [40] calculated that the input rate of SIC in Aridisols was 0.24 g C m^-2^ yr^-1^ in the Mojave Desert, and the accumulation rate of secondary carbonates ranged between 0.12-0.42 g C m^-2^ yr^-1^. These are in the same order of magnitude as in this study. However, this study highlighted that the rate of SDIC accumulation was high with a shorter residence time, which should be differentiated from SIC with a much lower input rate. 

### Concluding remarks

The key to estimating SIC and SDIC storage and dynamics in saline and alkaline soil profiles is understanding the processes of carbonate accumulation. We introduced a convenient way to indirectly determine SDIC content in these soils. Potential applications include assessment of the calculation of carbon fluxes and budgets in soil systems and investigation of the carbon storage potential of soils. Generally, through analyzing SDIC and SIC in saline and alkaline soil profiles at different depths, we found that more than 80% of the carbon was in storage below 1m. This is especially true for SIC and SDIC in saline and alkaline soils. Significantly, depth distribution is soil specific. In addition, soil contains more than 20% SDIC in soil inorganic carbon, and the comparison of residence time and input rate in the SDIC and SIC showed that the using SIC to estimate soil carbon turnover would considerably obscure an important fraction of the modern carbon cycle: namely the shorter residence and higher input rate of SDIC. Information on SIC and SDIC in the entire soil profile is crucial when assessing current regional, continental and global soil C storage and dynamics and in optimizing strategies to mitigate the accumulation of CO_2_ in the atmosphere.

## Supporting Information

File S1 **Supporting Tables.** Table S1, Mean pH and EC value changes and standard error in soil-water extract solution by centrifugation. Table S2, Mean SIC and SDIC content and standard error in saline and alkaline soil profiles. Table S3, Mean soil carbon density and standard error at different depths for saline and alkaline soils. Table S4, Mean residence times of SIC and SDIC and standard error at different soil layers in saline and alkaline soil profiles. Table S5, Mean rate of SIC and SDIC accumulation and standard error at different soil layers in saline and alkaline soil profiles.
(DOC)
