# Supplementary material for: Storage/Turnover Rate of Inorganic Carbon and Its Dissolvable Part in the Profile of Saline/Alkaline Soils
Source: PLoS One. 2013 Nov 29;8(11):e82029. doi: 10.1371/journal.pone.0082029 (PMC3843718; doi:10.1371/journal.pone.0082029)
Supplement: File S1 — Supporting Tables. Table S1, Mean pH and EC value changes and standard error in soil-water extract solution by centrifugation. Table S2, Mean SIC and SDIC content and standard error in saline and alkaline soil profiles. Table S3, Mean soil carbon density and standard error at different depths for saline and alkaline soils. Table S4, Mean residence times of SIC and SDIC and standard error at different soil layers in saline and alkaline soil profiles. Table S5, Mean rate of SIC and SDIC accumulation and standard error at different soil layers in saline and alkaline soil profiles. (DOC) [file pone.0082029.s001.doc]

**Supporting Information, Tables:**

**Table S1. Mean pH and EC value changes and standard error in soil-water extract solution by centrifugation.**

| leaching  time(n) | Saline soil | | | | | | Alkaline soil | | | | | | | |
| --- | --- | --- | --- | --- | --- | --- | --- | --- | --- | --- | --- | --- | --- | --- |
|  | 0.0-1.0 m | | 1.0-3.0 m | | 3.0-6.0 m | | 0.0-1.0 m | | 1.0-3.0 m | | 3.0-6.0 m | | 6.0-9.0 m | |
|  | Mean | se | Mean | se | Mean | se | Mean | se | Mean | se | Mean | se | Mean | se |
| EC value (ms/cm) | | | | | | | | | | | | | | |
| 1 | 3.54 | 0.55 | 1.32 | 0.24 | 0.45 | 0.02 | 0.68 | 0.13 | 0.53 | 0.06 | 0.47 | 0.16 | 0.78 | 0.06 |
| 2 | 0.73 | 0.07 | 0.42 | 0.04 | 0.23 | 0.02 | 0.47 | 0.09 | 0.21 | 0.03 | 0.16 | 0.03 | 0.23 | 0.02 |
| 3 | 0.28 | 0.03 | 0.25 | 0.02 | 0.18 | 0.01 | 0.16 | 0.02 | 0.09 | 0.01 | 0.08 | 0.01 | 0.10 | 0.00 |
| 4 | 0.15 | 0.01 | 0.17 | 0.02 | 0.14 | 0.01 | 0.08 | 0.01 | 0.07 | 0.00 | 0.07 | 0.01 | 0.08 | 0.00 |
| 5 | 0.10 | 0.01 | 0.13 | 0.02 | 0.10 | 0.01 | 0.07 | 0.00 | 0.05 | 0.00 | 0.06 | 0.00 | 0.07 | 0.01 |
| 6 | 0.09 | 0.00 | 0.11 | 0.02 | 0.09 | 0.01 | 0.05 | 0.00 | 0.04 | 0.00 | 0.05 | 0.00 | 0.05 | 0.01 |
| 7 | 0.06 | 0.00 | 0.07 | 0.01 | 0.06 | 0.00 | 0.05 | 0.00 | 0.04 | 0.00 | 0.04 | 0.00 | 0.04 | 0.00 |
| 8 | 0.05 | 0.00 | 0.06 | 0.01 | 0.05 | 0.00 | 0.05 | 0.00 | 0.04 | 0.00 | 0.04 | 0.00 | 0.04 | 0.00 |
| 9 | 0.05 | 0.00 | 0.05 | 0.00 | 0.04 | 0.00 | 0.05 | 0.00 | 0.04 | 0.00 | 0.04 | 0.00 | 0.04 | 0.00 |
| 10 | 0.04 | 0.00 | 0.05 | 0.00 | 0.04 | 0.00 |  |  |  |  |  |  |  |  |
| 11 | 0.04 | 0.00 | 0.05 | 0.00 | 0.04 | 0.00 |  |  |  |  |  |  |  |  |
| pH |  |  |  |  |  |  |  |  |  |  |  |  |  |  |
| 1 | 9.79 | 0.07 | 10.27 | 0.11 | 10.11 | 0.09 | 9.35 | 0.22 | 8.69 | 0.16 | 8.97 | 0.21 | 9.03 | 0.24 |
| 2 | 10.17 | 0.12 | 10.56 | 0.07 | 10.36 | 0.07 | 9.21 | 0.14 | 8.70 | 0.13 | 9.39 | 0.12 | 8.86 | 0.10 |
| 3 | 10.13 | 0.17 | 10.57 | 0.05 | 10.24 | 0.07 | 9.48 | 0.10 | 8.99 | 0.09 | 9.52 | 0.09 | 9.23 | 0.04 |
| 4 | 10.21 | 0.14 | 10.51 | 0.05 | 10.24 | 0.04 | 9.59 | 0.08 | 9.06 | 0.08 | 9.38 | 0.09 | 9.44 | 0.07 |
| 5 | 10.23 | 0.14 | 10.43 | 0.07 | 9.96 | 0.08 | 9.48 | 0.11 | 8.98 | 0.07 | 9.25 | 0.09 | 9.33 | 0.10 |
| 6 | 10.03 | 0.06 | 10.14 | 0.10 | 9.72 | 0.06 | 9.61 | 0.09 | 9.07 | 0.04 | 9.31 | 0.10 | 9.54 | 0.08 |
| 7 | 10.01 | 0.05 | 10.17 | 0.10 | 9.85 | 0.04 | 9.63 | 0.07 | 9.03 | 0.05 | 9.30 | 0.14 | 9.57 | 0.04 |
| 8 | 10.00 | 0.04 | 10.01 | 0.11 | 9.80 | 0.04 | 9.60 | 0.06 | 8.98 | 0.06 | 9.32 | 0.05 | 9.56 | 0.02 |
| 9 | 9.85 | 0.03 | 9.97 | 0.11 | 9.79 | 0.02 | 9.52 | 0.05 | 9.05 | 0.05 | 9.34 | 0.08 | 9.48 | 0.06 |
| 10 | 9.90 | 0.04 | 9.98 | 0.09 | 9.77 | 0.03 |  |  |  |  |  |  |  |  |
| 11 | 9.90 | 0.01 | 9.83 | 0.12 | 9.59 | 0.04 |  |  |  |  |  |  |  |  |

**Table S2. Mean SIC and SDIC content and standard error in saline and alkaline soil profiles.**

| Depth(m) | saline soil (g kg^-1^) | | | | alkaline soil (g kg^-1^) | | | |
| --- | --- | --- | --- | --- | --- | --- | --- | --- |
|  | SIC | | SDIC | | SIC | | SDIC | |
|  | Mean | se | Mean | se | Mean | se | Mean | se |
| 0.0-0.2 | 8.31 | 0.25 | 1.10 | 0.07 | 5.44 | 0.11 | 1.80 | 0.02 |
| 0.2-0.4 | 9.59 | 0.35 | 2.67 | 0.17 | 5.46 | 0.16 | 1.85 | 0.15 |
| 0.4-0.6 | 8.88 | 0.34 | 3.59 | 0.17 | 5.26 | 0.07 | 1.71 | 0.09 |
| 0.6-0.8 | 9.13 | 0.34 | 3.77 | 0.10 | 4.90 | 0.10 | 1.63 | 0.10 |
| 0.8-1.0 | 8.87 | 0.49 | 3.36 | 0.15 | 4.66 | 0.09 | 1.22 | 0.27 |
| 1.0-1.5 | 8.91 | 0.41 | 2.52 | 0.08 | 4.03 | 0.09 | 1.00 | 0.16 |
| 1.5-2.0 | 6.14 | 0.27 | 1.50 | 0.08 | 3.90 | 0.10 | 1.10 | 0.09 |
| 2.0-2.5 | 8.11 | 0.54 | 2.26 | 0.23 | 4.26 | 0.09 | 1.19 | 0.12 |
| 2.5-3.0 | 11.55 | 0.46 | 3.37 | 0.06 | 3.73 | 0.17 | 0.85 | 0.11 |
| 3.0-4.0 | 13.92 | 0.75 | 2.92 | 0.24 | 5.39 | 0.23 | 1.25 | 0.21 |
| 4.0-5.0 | 13.34 | 0.32 | 2.87 | 0.14 | 3.89 | 0.13 | 0.93 | 0.16 |
| 5.0-6.0 | 12.65 | 0.65 | 2.53 | 0.16 | 4.23 | 0.27 | 1.12 | 0.25 |
| 6.0-7.0 |  |  |  |  | 7.06 | 0.13 | 0.99 | 0.07 |
| 7.0-8.0 |  |  |  |  | 6.53 | 0.26 | 0.64 | 0.07 |
| 8.0-9.0 |  |  |  |  | 5.03 | 0.13 | 0.25 | 0.08 |

**Table S3**. Mean soil carbon density and standard error at different depths for saline and alkaline soils.

| Depth(m) | saline soil (kg m^-2^) | | | | alkaline soil (kg m^-2^) | | | |
| --- | --- | --- | --- | --- | --- | --- | --- | --- |
|  | SIC | | SDIC | | SIC | | SDIC | |
|  | Mean | se | Mean | se | Mean | se | Mean | se |
| 0.0-1.0 | 11.38 | 4.43 | 3.84 | 0.62 | 7.44 | 0.93 | 2.37 | 0.31 |
| 1.0-3.0 | 26.34 | 12.03 | 7.33 | 0.98 | 11.06 | 1.42 | 2.87 | 0.39 |
| 3.0-6.0 | 62.25 | 23.18 | 13.03 | 2.68 | 20.18 | 4.75 | 4.93 | 1.13 |
| 6.0-9.0 |  |  |  |  | 26.59 | 5.27 | 2.7 | 0.27 |

**Table S4**. Mean residence times of SIC and SDIC and standard error at different soil layers in saline and alkaline soil profiles.

| Depth(m) | saline soil (year) | | | | alkaline soil (year) | | | |
| --- | --- | --- | --- | --- | --- | --- | --- | --- |
|  | SIC | | SDIC | | SIC | | SDIC | |
|  | Mean | se | Mean | se | Mean | se | Mean | se |
| 0.0-1.0 | 8477 | 92 | 130 | 40 | 9456 | 66 | 484 | 32 |
| 1.0-3.0 | 10971 | 68 | 312 | 43 | 11923 | 53 | 1470 | 43 |
| 3.0-6.0 | 11688 | 82 | 1781 | 52 | 19121 | 80 | 3162 | 46 |
| 6.0-9.0 |  |  |  |  | 20950 | 105 | 1892 | 40 |

**Table S5**. Mean rate of SIC and SDIC accumulation and standard error at different soil layers in saline and alkaline soil profiles.

| Depth(m) | saline soil (g C m^-2^ year^-1^) | | | | alkaline soil (g C m^-2^ year^-1^) | | | |
| --- | --- | --- | --- | --- | --- | --- | --- | --- |
|  | SIC | | SDIC | | SIC | | SDIC | |
|  | Mean | se | Mean | se | Mean | se | Mean | se |
| 0.0-1.0 | 1.34 | 0.52 | 29.54 | 4.77 | 0.79 | 0.10 | 4.90 | 0.64 |
| 1.0-3.0 | 2.40 | 1.10 | 23.49 | 3.14 | 0.93 | 0.12 | 1.95 | 0.27 |
| 3.0-6.0 | 5.33 | 1.98 | 7.58 | 1.56 | 1.06 | 0.25 | 1.56 | 0.36 |
| 6.0-9.0 |  |  |  |  | 1.27 | 0.25 | 1.43 | 0.14 |
